# Population pharmacokinetics of vancomycin in very low birth weight neonates

**DOI:** 10.3389/fped.2023.1093171

**Published:** 2023-03-30

**Authors:** Abdullah Alsultan, Manea Fares Al Munjem, Khulood Mohammed Atiq, Zekra Kamel Aljehani, Hessa Al Muqati, Abdullah Almohaizeie, Dalia Ahmed Ballal, Tahani Makki Refaei, Majed Al Jeraisy, Abdulmohsen Assiri, Manal Abouelkheir

**Affiliations:** ^1^ Department of Clinical Pharmacy, College of Pharmacy, King Saud University, Riyadh, Saudi Arabia; ^2^ King Khalid Hospital, Najran, Saudi Arabia; ^3^ Prince Sultan Military Medical City, Riyadh, Saudi Arabia; ^4^ Pharmaceutical Care Division, King Faisal Specialist Hospital and Research Center, Jeddah, Saudi Arabia; ^5^ Pharmacy Department, King Abdulaziz Medical City, Riyadh, Saudi Arabia; ^6^ Pharmaceutical Care Division, King Faisal Specialist Hospital and Research Center, Riyadh, Saudi Arabia; ^7^ Pharmaceutical Care Administration, Armed Forces Hospital Southern Region, Khamis Mushait, Saudi Arabia; ^8^ King Saud bin Abdulaziz University for Health Sciences, Riyadh, Saudi Arabia; ^9^ King Abdullah International Medical Research Center, Riyadh, Saudi Arabia; ^10^ Department of Clinical Pharmacy, Faculty of Pharmacy, Misr International University, Cairo, Egypt

**Keywords:** pharmacokenetics, very low birth weigh neonates, vancomycin, infectious disease, pharmacodynamic

## Abstract

**Introduction:**

Vancomycin dosing in very low birth weight (VLBW) neonates is challenging. Compared with the general neonatal population, VLBW neonates are less likely to achieve the vancomycin therapeutic targets. Current dosing recommendations are based on studies of the general neonatal population, as only a very limited number of studies have evaluated vancomycin pharmacokinetics in VLBW neonates. The main aim of this study was to develop a vancomycin population pharmacokinetic model to optimize vancomycin dosing in VLBW neonates.

**Methods:**

This multicenter study was conducted at six major hospitals in Saudi Arabia. The study included VLBW neonates who received vancomycin and had at least one vancomycin serum trough concentration measurement at a steady state. We developed a pharmacokinetic model and performed Monte Carlo simulations to develop an optimized dosing regimen for VLBW infants. We evaluated two different targets: AUC_0–24_ of 400–600 or 400–800 µg. h/mL. We also estimated the probability of trough concentrations >15 and 20 µg/mL.

**Results:**

In total, we included 236 neonates, 162 in the training dataset, and 74 in the validation dataset. A one-compartment model was used, and the distribution volume was significantly associated only with weight, whereas clearance was significantly associated with weight, postmenstrual age (PMA), and serum creatinine (Scr).

**Discussion:**

We developed dosing regimens for VLBW neonates, considering the probability of achieving vancomycin therapeutic targets, as well as different toxicity thresholds. The dosing regimens were classified according to PMA and Scr. These dosing regimens can be used to optimize the initial dose of vancomycin in VLBW neonates.

## Introduction

Late-onset neonatal sepsis is a major cause of morbidity and mortality in newborns worldwide, with a mortality rate of approximately 15% in very low birth weight (VLBW) neonates (birth weight <1,500 g) (1–3). Vancomycin is a glycopeptide antibiotic frequently used to treat nosocomial gram-positive infections in neonates when methicillin-resistant *Staphylococcus aureus* (MRSA) or coagulase-negative staphylococci (MRCONS) are suspected. Vancomycin is considered one of the most common antimicrobial agents used in neonatal intensive care units (NICU) because of increased mortality and morbidity rates due to neonatal sepsis, compared with sepsis in children (4, 5). The optimal vancomycin dose should target an area under curve/minimum inhibitory concentration (AUC_0−24_/MIC) of 400 µg.h/ml, while minimizing higher AUCs_0−24_/MIC (>800 µg.h/ml) and troughs (>20 µg/ml) (6–10). The new IDSA treatment guidelines recommend targeting an AUC_0−24_ of 400–600 µg.hr/ml (assuming an MIC of 1 µg/ml) and no longer recommends trough guided monitoring. Although vancomycin has been used in clinical practice for more than 50 years, dosing in neonates remains challenging for many reasons. Most notably, these include a lack of consensus on the optimal dosing and a narrow therapeutic index (5, 10, 11–14). In addition, the rapidly changing pharmacokinetics in neonates are mainly related to changes in body water and organ maturation. Previous studies have reported that the major predictors of vancomycin pharmacokinetics in neonates include body weight, maturation [as indicated by postmenstrual age (PMA), gestational age (GA), and postnatal age (PNA)], and serum creatinine (Scr) (11, 12, 15).

Optimizing vancomycin dosing is even more challenging in VLBW neonates than in the general neonatal population and older pediatric patients. VLBW neonates exhibit limited renal elimination and higher extracellular fluid volume compared to normal weight neonates (16, 17). In our previous study, we found that VLBW neonates were less likely to achieve a vancomycin therapeutic target of an AUC_0−24_/MIC > 400 µg.h/ml compared to neonates born with a larger body weight (18). Similar findings were observed in the study by Stone et al. (19). This indicates that current dosing recommendations are not optimal for the VLBW neonates and there is a need for specific dosing regimens in this patient population. Most published pharmacokinetic models are in the general neonatal population. To date, only two studies evaluating vancomycin pharmacokinetics and dosing in VLBW neonates have been published (16, 17). Both of these studies included a very small sample size (<20 patients). Therefore, pharmacokinetic models must be further explored in this unique population.

Nevertheless, early optimization of vancomycin dosing to rapidly achieve adequate antibiotic exposure is extremely important. This will increase efficacy, minimize toxicity, and avoid bacterial resistance, particularly when treating invasive MRSA infections. Therefore, the main aim of this study was to develop a vancomycin population pharmacokinetic model to optimize dosing in VLBW neonates.

## Methods

### Patients and data collection

This multicenter observational study was conducted in six major hospitals in Saudi Arabia. Data were retrospectively collected from the electronic medical records of the following hospitals: King Saud University Medical City in Riyadh, King Faisal Specialist Hospital in Riyadh, National Guard Hospital in Riyadh, King Faisal Specialist Hospital in Jeddah, Prince Sultan Military Medical City in Riyadh, and Armed Forces Hospital Southern Region in Abha. Data were retrieved for VLBW neonates (0–30 days old) who were admitted to the NICU and received vancomycin for proven or suspected MRSA or MRCONS infections. Patients were included if they received vancomycin for more than 48 h and had at least one vancomycin serum trough concentration measurement in a steady state. The collected data included PNA, PMA, weight, Scr, vancomycin dose, frequency and concentrations. For vancomycin initial dosing recommendations, online pediatric and neonatal Lexi-Drugs® (Lexicomp, Ohio, USA) (20) was the main neonatal dosing reference at National Guard Hospital, whereas all other participating hospitals relied mainly on Micromedex NeoFax (IBM Corp., Armonk, NY, United States) (21) (Table 1). Subsequent dose adjustments were guided by the vancomycin trough concentrations. In King Saud University Medical City, both vancomycin peak and trough concentrations were obtained for therapeutic drug monitoring. In all other hospitals, the practice was to collect trough concentrations only. Peak samples were collected 1 h after the end of infusions, and trough concentrations were collected 30 min before the next dose in a steady state. Exclusion criteria were: renal failure, receiving hemodialysis, missing dosing information and those sampled before the steady state were excluded. The study was IRB approved in all six participating centers.

**Table 1:** Vancomycin dosing regimens from neonatal dosing references commonly used in participating hospitals.

Reference	PMA^a^ (weeks)	PNA^b^ (days)	Dose (mg/kg/dose)	Interval (hours)
**NeoFax**	≤29	≤14	10–15	18
		>14		12
	30–36	≤14	10–15	12
		>14		8
	37–44	≤7	10–15	12
		>7		8
	≥45	All	10–15	6
**Lexicomp** Age directed dosing	PMA^a^ (weeks)	PNA^b^ (days)	Dose (mg/kg/dose)	Interval (hours)
	≤29	≤21	15	18
		> 21	15	12
	30 to <37	≤14	15	12
		>14	15	8
	37–45	≤7	15	12
		>7	15	8
**Lexicomp** Kidney function based dosingd	GA^c^ (weeks)	Scr (mg/dl)		
	≤28	<0.5	15	12
		0.5–0.7	20	24
		0.8–1	15	24
		1.1–1.4	10	24
		>1.4	15	48
	>28	<0.7	15	12
		0.7–0.9	20	24
		1–1.2	15	24
		1.3–1.6	10	24
		>1.6	15	48

^a^ PMA, postmenstrual age. PMA is gestational age plus postnatal age.

^b^ PNA, postnatal age.

^c^ GA, Gestational age.

^d^ Kidney function based dosing: a loading dose of 20 mg/kg is recommended. It was designed to achieve target trough concentrations of 5 to 10 µg/ml.

### Analytical assay

Three different validated methods were used for the analytical assay, as each hospital had a different system. One hospital used a chemiluminescent microparticle immunoassay with an Architect i4000SR immunoassay analyzer (Abbott), two hospitals used the kinetic interaction of microparticles in solution (KIMS) with a Cobas c system (Roche/Hitachi), and three hospitals used the homogeneous particle-enhanced turbidimetric inhibition immunoassay (PETINIA) with an Alinity c analyzer (Abbott). The assay range for the Architect immunoassay is from 0.5 to 83 µg/ml, the range for the KIMS assay is 4–80 µg/ml, and the range for the PETINIA is 1.4–100 µg/ml. For the Scr assays, four centers used the Jaffe method, and two centers used the enzymatic method. The following formula was used to convert the enzymatic assay to the Jaffe method (22):

$$Jaffe\;concentration=\;0.122+\frac{Enzymatic\;concentration}{1.05}$$

### Population pharmacokinetics

We split the data randomly into the training (70%) and validation (30%) data sets. Data splitting was performed using R statistical software. Pharmacokinetic modeling was performed using the Monolix software (2020R1) with the stochastic approximation expectation maximization algorithm.

### Base model

First, we developed a structural model for vancomycin. This included testing one- and two-compartment models with linear and nonlinear elimination. For the between-subject variability in pharmacokinetic parameters, we used the following lognormal distribution:

$$p_{i}=p.\exp(\eta_{i}),$$

where $p_{i}$ is the individual pharmacokinetic estimate for the *i*^th^ individual, *p* is the median value of the pharmacokinetic parameter, and $\eta_{i}$ is the standard deviation for the *i*^th^ individual with a mean of 0 and variance *ω*^2^. For residual unexplained variability, we tested different statistical models, including the constant, proportional, and combined error models. As different assays were used for vancomycin, we tested having different residual variability models accordingly.

### Covariates

After we developed the base model, we evaluated the effect of covariates on pharmacokinetic parameters. We tested the effects of postnatal age, gestational age, PMA, weight, height, sex, Scr, presence of congenital heart disease, and VLBW vs. extremely low birth weight (ELBW) on all pharmacokinetic parameters. Covariates were tested by plotting the *post hoc* individual pharmacokinetic parameters (empirical Bayesian estimates) against covariates to identify possible correlations. Testing was performed stepwise using a likelihood ratio rest. The significance level was set at *p* < 0.05. The effect of continuous covariates was modeled using a power function as follows:

$$P_{i}=P\;*\;{\left(\frac{X_{i}}{X}\right)}^{\beta },$$

where $P_{i}$ is the individual pharmacokinetic parameter for the *i*^th^ individual, *P* is the typical value of the pharmacokinetic parameter in the population, $X_{i}$ is the covariate value for the *i*^th^ individual, and $X_{i}$ is the population mean of the covariate. The exponents for the effects of weight on total body clearance (Cl) and the volume of distribution (V) were fixed at 0.75 and 1, respectively (23–25). To determine the effect of age on Cl, we tested both power, linear, and sigmoidal functions to account for maturation (25, 26). The sigmoidal function is expressed as follows:

$$F_{PMA}=\frac{PMA^{hill}}{PMA^{hill}+TMA_{50}^{hill}},$$

where *hill* is the Hill coefficient for clearance, and TMA_50_ is the PMA, where Cl is 50% of the mature value.

### Model evaluation

Model evaluation and selection were guided by the objective function value, physiological plausibility, and standard diagnostic plots. The relative standard error of the pharmacokinetic estimates was also assessed, with an optimal value of <30%.

### Validation

The final population pharmacokinetic model was evaluated using the validation dataset. Model performance was assessed using simulation diagnostics, Visual Predictive Check (VPC), and Normalized Prediction Distribution Error (NPDE) (27). The null hypothesis for the NPDE is that it follows a normal distribution, with a mean of 0 and a standard deviation of 1. The VPC is based on 1,000 dataset simulations, followed by a comparison between the observed data and the simulated concentrations.

### Simulation

The final model was used to perform Monte Carlo simulations to determine the optimal vancomycin dose. We evaluated various doses ranging from to 10–20 mg/kg in 2.5 mg/kg increments every 6, 8, 12, 18, and 24 h. The final population pharmacokinetic model with the distribution of the pharmacokinetic parameters was embedded into the “Simulx” package in R. Simulx is part of the “mlxr” package in R and can be used to simulate the time concentration profile for different pharmacokinetic models (Lixoft, Antony, France, http://simulx.webpopix.org/). To ensure that the distribution of the significant covariates was identical to our patient population, we replicated our dataset 40 times (40 × 236 = 9,440). We simulated the time-concentration profiles for all 9,440 cases. We evaluated two targets in the simulations: (1) AUC_0−24_ of 400–600 µg.h/ml, as recommended in the IDSA treatment guidelines, (10) and (2) the less conservative target of AUC_0−24_ of 400–800 µg.h/ml, based on a prior study in pediatrics demonstrating increased nephrotoxicity for AUC_0−24_ > 800 µg.h/ml (28). We also estimated the probability of both trough levels >5 µg/ml and 20 µg/ml for each dosing regimen. Here, we considered troughs as the secondary marker for nephrotoxicity, as higher trough concentrations are also associated with nephrotoxicity (29–31). Prior studies in adults indicate risk of nephrotoxicity is approximately 15% for troughs between 15 and 20 µg/ml and increases to approximately 30% for troughs >20 µg/ml (30). Therefore, troughs >20 µg/ml should be always avoided, while troughs >15 µg/ml should be minimized if possible, as some patients can achieve a vancomycin therapeutic target of an AUC_0−24_ 400–600 µg.h/ml at troughs <15 µg/ml (12, 30–32).

## Results

In total, we included 236 patients: 162 in the training dataset, and 74 in the validation dataset. The total number of observations was 214 for the training dataset, and 97 for the validation dataset. Out of the 236 patients 36% had a culture confirmed infection. Approximately 58% of our patient population were extremely low birth weight (59% in the training data set and 55% in the validation data set). Both the training and validation data sets were similar in terms of bodyweight, PMA and Scr. The full baseline demographics of both datasets are shown in Table 2.

**Table 2:** Baseline demographics.

	Training data set (*N* = 162)	Validation data set (*N* = 74)
	Mean (sd, minimum-maximum)	Mean (sd, minimum-maximum)
Age (days)	10.7 (7.5, 1–30)	11.8 (9.0, 2–30)
PMA (weeks)	29.8 (3.15, 22–39)	30.7 (3.4, 24–42)
GA (weeks)	28.0 (2.9, 22–35)	28.4 (2.8, 23–38)
Gender	Male: 45%	Male: 49%
	Female: 32%	Female: 34%
	Missing: 22%	Missing: 18%
ELBW	59%	55%
Birth weight (Kg)	0.95 (0.27, 0.46–1.5)	1.0 (0.26, 0.5–1.5)
Weight (Kg)	1.0 (0.29, 0.46–1.7)	1.1 (0.3, 0.5–2.2)
Scr (mg/dl)	0.65 (0.22, 0.2–1.5)	0.62 (0.23, 0.15–1.4)
Total daily dose (mg/kg)	22 (8, 7.5–55)	24.1 (11.5, 9–68)
Congenital heart disease	26%	37%
Culture confirmed infections	60 (37%)	25 (33%)

### Population pharmacokinetics

Vancomycin pharmacokinetic data were best described using a one-compartment model with linear elimination and a proportional error model. Significant covariates identified were weight for volume of distribution (V) and weight, Scr, and PMA for clearance (Cl). Bodyweight was scaled to the median bodyweight of 0.93 kg, and the exponents for the effect of weight on V and Cl were fixed at 1 and 0.75 (23, 25, 26). The effect of PMA was best described using a sigmoidal function. The typical Cl value for a VLBW neonate weighing 0.93 kg, PMA equal to 26 weeks, and Scr of 0.6 mg/dl was 0.09 L/h, while the typical V value for a VLBW neonate weighing 0.93 kg was 0.81 L. The formulas below describe the predicted values of Cl and V:

$$V=0.81*\left(\frac{Weight}{0.93}\right)\;$$

$$Cl=0.09*{\left(\;\;\frac{Weight}{0.93}\;\;\right)}^{\;\;0.75}*{\left(\;\;\frac{0.6}{serum\;\;creatinine\;}\;\;\right)}^{\;\;0.48}*\frac{\mathrm{PM}A^{4.42}}{\mathrm{PM}A^{4.42}+\;26.3^{4.42}}$$

**Figure 1:**
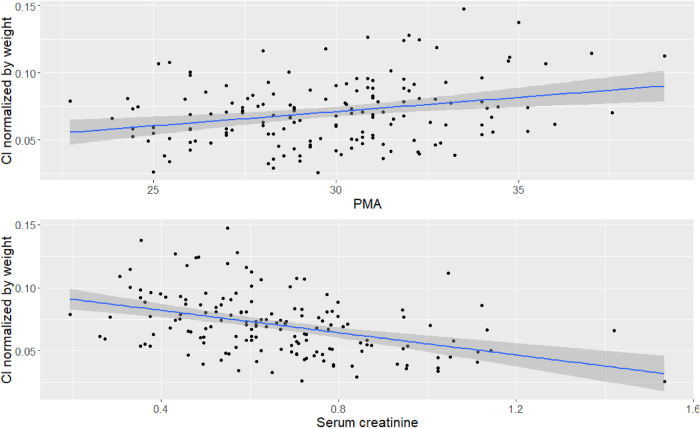
Correlation between covariates and Cl. Top figure is correlation between PMA and Cl normalized by bodyweight, bottom figure is correlation between Scr and Cl normalized by bodyweight.

**Figure 2:**
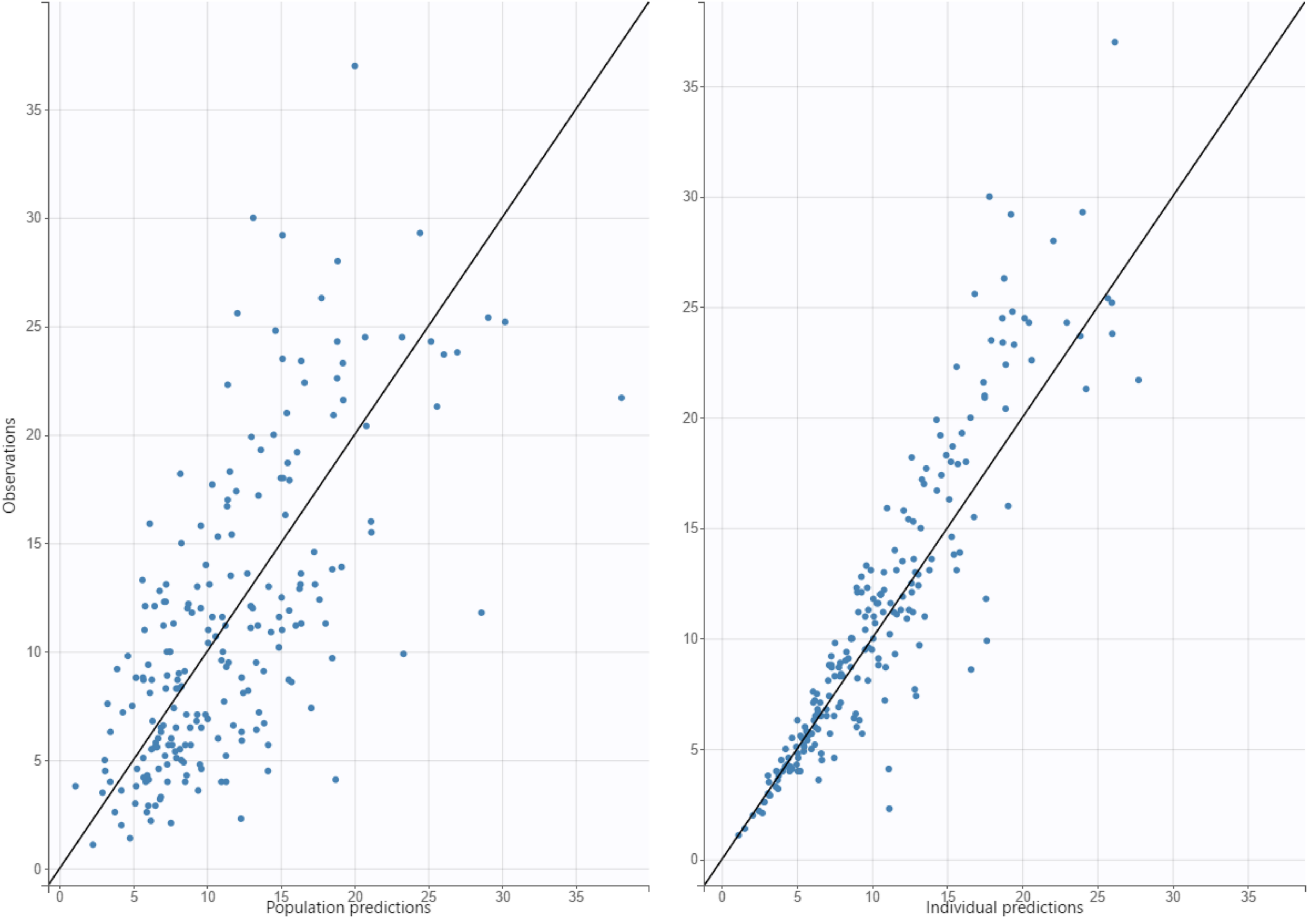
Diagnostic plot for observed concentrations vs. predicted concentrations, figure on left is population predictions, figure on right is individual predictions.

**Figure 3:**
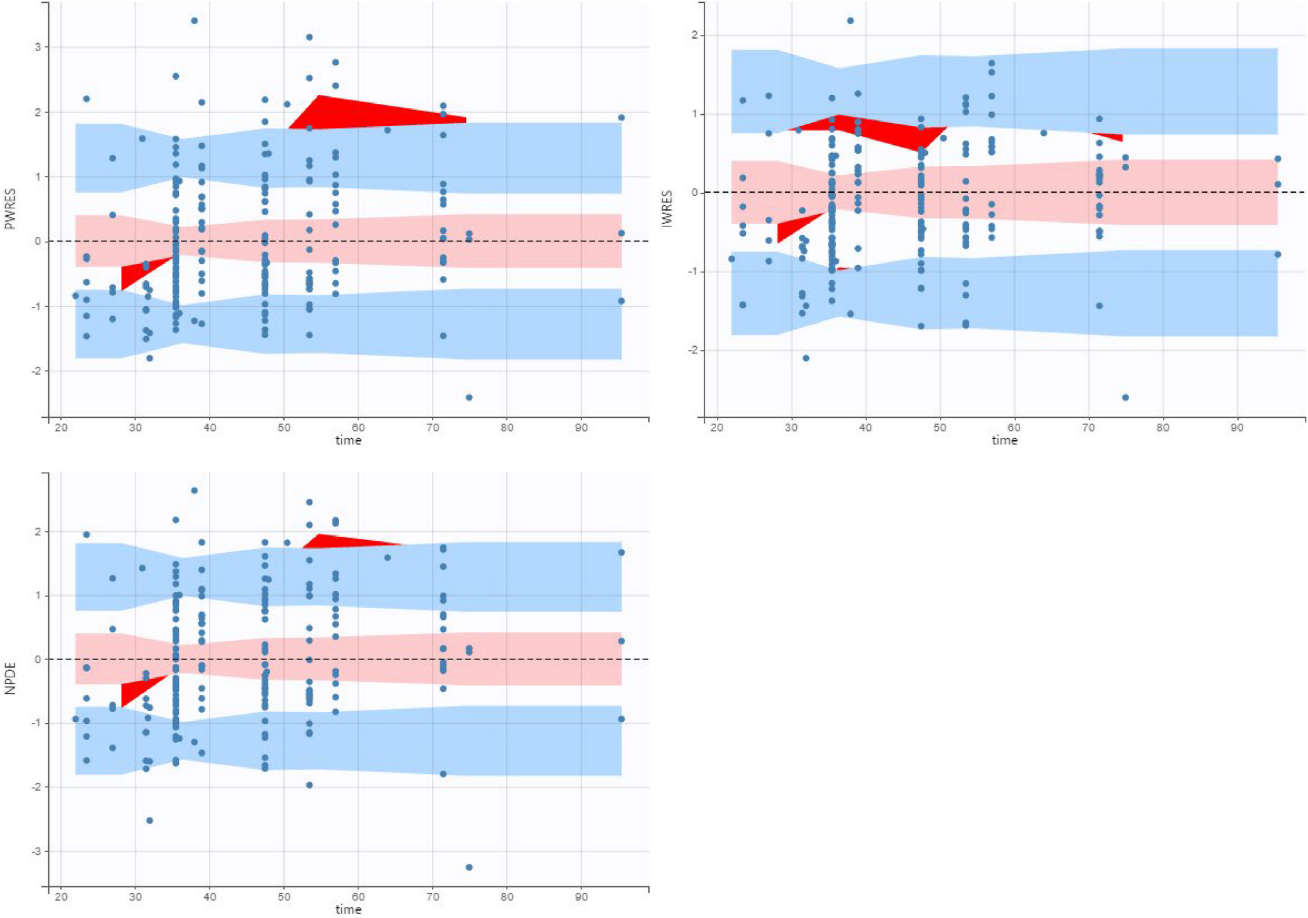
Population weighed residuals (top left), individual weighed residuals plot (top right) and normalized prediction distribution error plot (bottom left) for the final population pharmacokinetic model.

**Figure 4:**
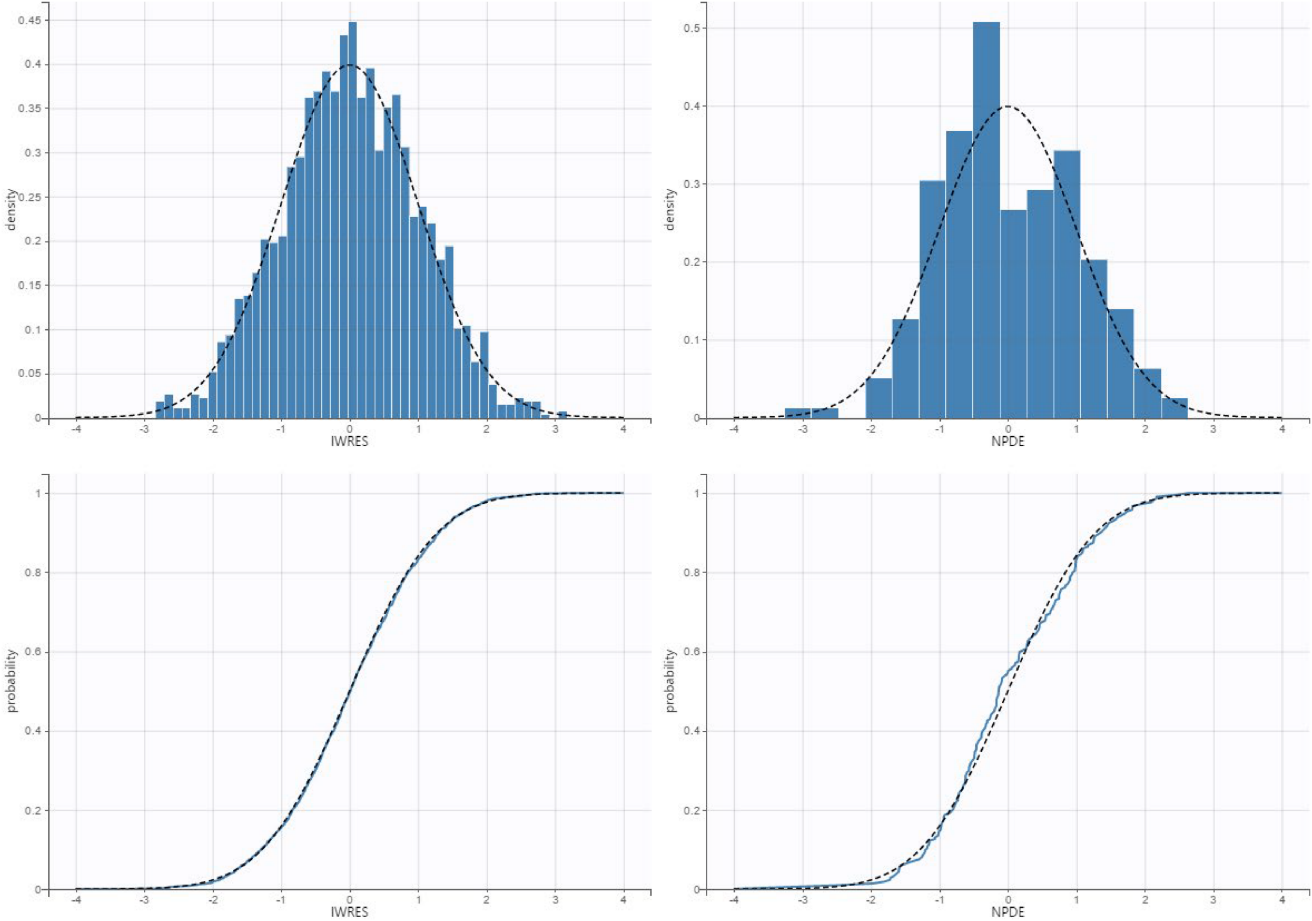
Qq plot and histogram of the individual weighed residuals (left) and normalized prediction errors (right).

Figure 1 shows the correlation between Cl normalized by body weight vs. Scr and PMA. The results of the final model are presented in Table 3. Diagnostic plots are shown in Figures 2–4.

**Table 3:** Pharmacokinetic estimates for the final population pharmacokinetic model.

	Estimate	RSE%
V (L)	0.81	6.6%
IIV V	24%	26%
Cl (L/hr)	0.09	11%
IIV Cl	28%	11%
Hill coefficient for clearance	4.42	19%
TMA_50_ (weeks)	26.3	7%
Exponent of Scr on Cl	0.48	19%
Residual variability b	0.3	9.9%

V, volume of distribution; IIV, interindividual variability; Cl, total body clearance; TMA, is the PMA at which CL is 50% of the mature value; Scr, serum creatinine; b, proportional error; RSE, relative standard error.

### Model validation

**Figure 5:**
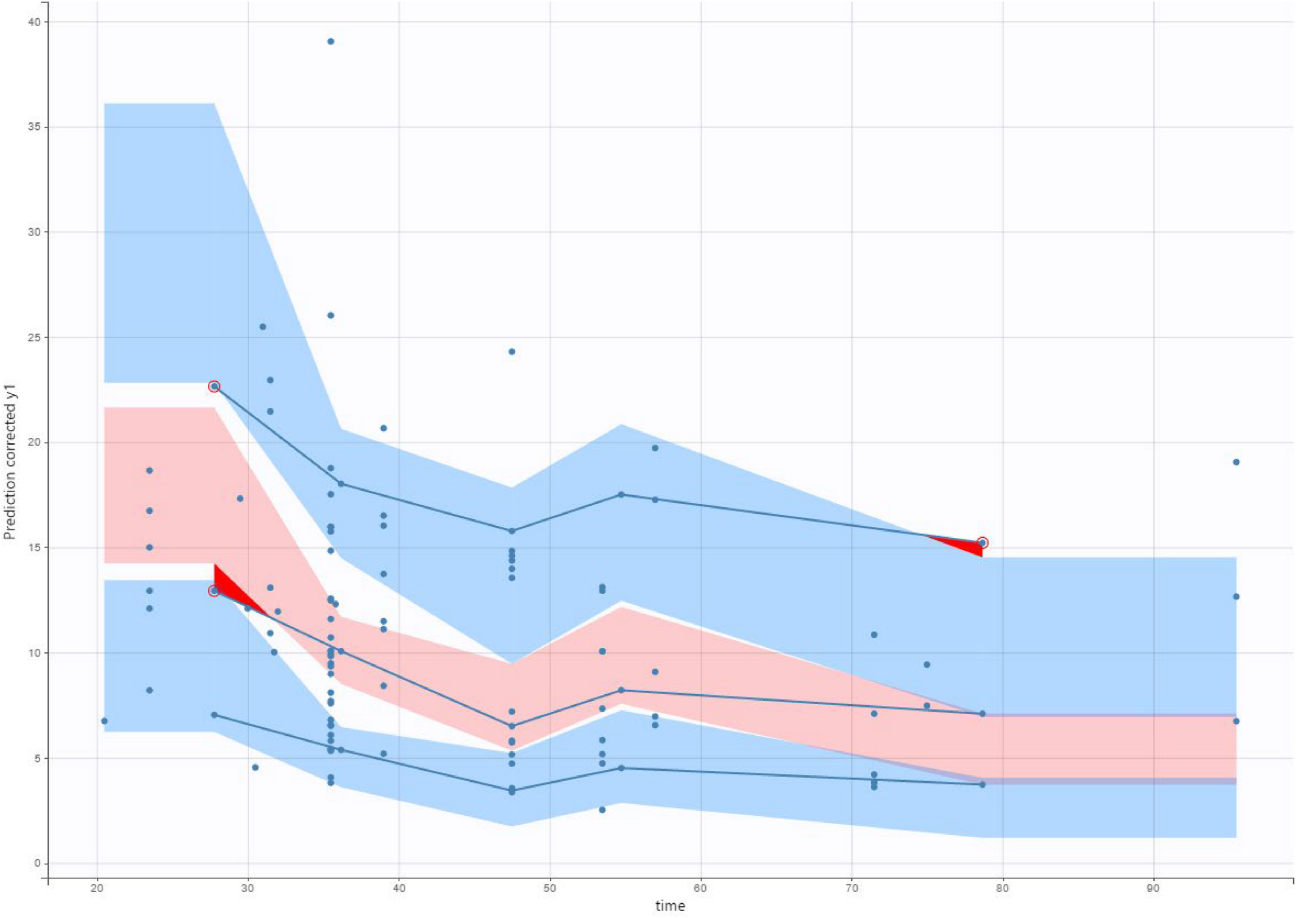
Visual predictive check (VPC) of our final model applied to the validation data set. The solid lines represent the 10th, 50th and 90th percentiles of observed data. The shaded regions represent the 90% confidence interval around the 10th, 50th and 90th percentiles of simulated data. The circles are observed concentrations.

**Figure 6:**
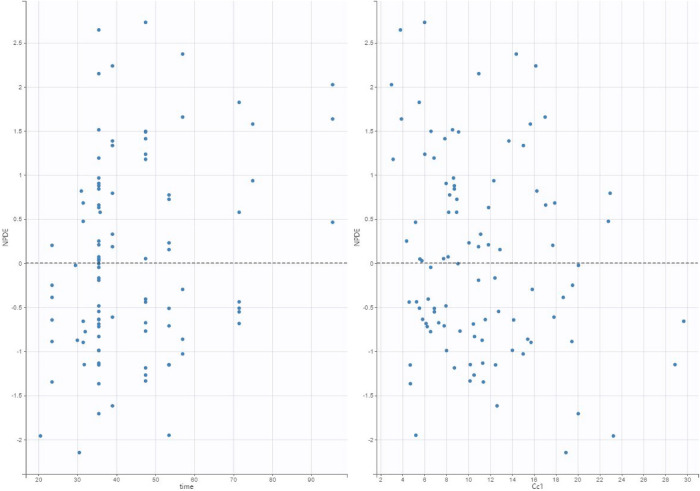
Normalized prediction distribution error (NPDE) plot of our final model applied to the validation data set.

The validation dataset contained 97 observations from 74 participants. The diagnostic plots are shown in Figures 5 (VPC) and Figure 6 (NPDE). For the VPC, the observed and predicted medians and percentiles were similar. The mean NPDE was 0.043, and the standard deviation was 1.1, indicating that the model had limited bias and captured most of the data variability.

### Simulation

Simulations were performed for various dosing regimens based on the final population pharmacokinetic model. The simulations were performed for different age and Scr groups as follows: PMA above and below 29 weeks and Scr < 0.6, from 0.6–0.9, and >0.9–1.2 mg/dl, for a total of six groups. There were only five patients with Scr > 1.2 mg/dl; therefore, the simulations were capped at 1.2 mg/dl. The range for Scr, PMA and weight in the simulation data set was: Scr from 0.2–1.2 mg/dl, PMA from 22 to 42 weeks and for bodyweight was from 0.46 to 2.2 kg. Table 4 shows the simulations for selected dosing regimens; we only presented the doses that meet these criteria: probability of AUC_0−24_ > 400 µg/h/ml is >60%, and probability of AUC_0−24_ > 800 µg.h/ml or trough >20 µg/ml is <10% to avoid nephrotoxicity. For each group, we identified 2–4 dosing regimens that meet these criteria. Dose selection would depend on the selected target: the narrower target of AUC_0−24_ which is equal to 400–600 µg.h/ml, or the wider target of AUC_0−24_ which is equal to 400–800 µg.h/ml.

**Table 4:** Summary statistics of the predicted AUC and trough concentrations and Monte Carlo simulations for various dosing regimens of vancomycin in VLBW neonates.

PMA (wks)	Scr (mg/dl)	Dose	AUC_0−24_ mean (sd) µg.h/ml	Trough mean (sd) µg/ml	AUC_0−24_ 400–600 µg.h/ml	AUC_0−24_ 400–800 µg.h/ml	Trough > 15 µg/ml
≤29	<0.6	15 mg/kg q12h	440 (107)	10.5 (3.43)	53%	61%	11%
		17.5 mg/kg q12h^a^	513 (125)	12.2 (4)	62%	80%	23%
		20 mg/kg q12h	586 (143)	14 (4.6)	50%	84%	38%
	0.6–0.9	17.5 mg/kg q18h	451 (116)	10.2 (3.8)	55%	65%	10%
		20 mg/kg q18h	515 (133)	11.6 (4.3)	58%	78%	20%
		15 mg/kg q12h^a^	523 (118)	13.3 (3.64)	63%	84%	28%
	> 0.9 to1.2	15 mg/kg q18h	450 (108)	10.8 (3.5)	56%	70%	27%
		17. 5 mg/kg q18h^a^	525 (127)	12.5 (4.1)	60%	83%	27%
		20 mg/kg q24h	480 (127)	10.3 (4.2)	56%	70%	13%
>29	<0.6	12.5 mg/kg q8h	452 (115)	12.5 (1)	56%	66%	26%
		20 mg/kg q12h	494 (131)	11 (4.3)	56%	74%	17%
	0.6–0.9	15 mg/kg q12h	453 (106)	11 (3.4)	57%	69%	12%
		17.5 mg/kg q12h^a^	528 (124)	12.8 (4)	60%	82%	28%
	> 0.9 to1.2	17.5 mg/kg q12h	440 (116)	9.8 (3.8)	51%	63%	3%
		20 mg/kg q18h^a^	503 (133)	11.2 (4.3)	76%	76%	18%
		15 mg/kg q12h^a^	513 (119)	12.9 (3.7)	58%	83%	27%

For all doses, the probability of an AUC_0−24_ > 800 µg. h/ml and/or trough >20 µg/ml is less than 10%.

^a^ The selected recommended dose to achieve the therapeutic target of an AUC0-24 of 400–600 µg.h/ml in more than 60% of patients or to achieve the wider range of 400–800 µg.h/ml in more than 80% of patients.

Table 5 shows the recommended optimal dose depending on the selected target (400–600 or 400–800 µg.h/ml). The recommend doses to maintain an AUC_0−24_ of 400–600 µg.h/ml are expected to achieve the therapeutic target in more than 60% of patients. If we select the wider range of 400–800 µg.h/ml, the recommended doses would achieve the target in more than 80% of patients. However, the 400–800 µg.h/ml target would require higher doses and may increase the probability of elevated trough concentrations and AUCs. The only exception was for neonates with PMA > 29 weeks and Scr < 0.6 mg/dl, the highest achieved probabilities were 56% for the target AUC_0−24_ of 400–600 µg.h/ml and 74% for the target AUC_0−24_ of 400–800 µg.h/ml with the selected dosing regimen.

**Table 5:** Recommended doses using our model depending on the target selected.

PMA (weeks)	Scr (mg/dl)	Recommended dose if targeting AUC_0−24_ 400–600 µg. h/ml	Recommended dose if targeting AUC_0−24_ 400–800 µg. h/ml
≤29	<0.6	17.5 mg/kg q12h	17.5 mg/kg q12h
	0.6–0.9	15 mg/kg q12h	15 mg/kg q12h
	0.9–1.2	17.5 mg/kg q18h	17.5 mg/kg q18h
>29	<0.6	12.5 mg/kg q8h	20 mg/kg q12h
	0.6–0.9	17.5 mg/kg q12h	17.5 mg/kg q12h
	0.9–1.2	20 mg/kg q18h	15 mg/kg q12h

## Discussion

Vancomycin, a drug with a narrow therapeutic window, is commonly used to treat life-threatening infections in neonates, such as MRSA. Previous clinical studies have demonstrated that vancomycin efficacy is maximized at AUC_0−24_/MIC > 400 µg.h/ml (33–35). Vancomycin dosing in VLBW patients is challenging. In this study, we developed a population pharmacokinetic model and identified the optimal initial dose for the VLBW neonate population. The significant covariates identified in our analysis were weight for V and weight, Scr, and PMA for Cl, consistent with prior studies in neonates (36). Also, our estimates for V and Cl are within range of previous studies (36).

The most commonly used tertiary dosing references present vancomycin dosing recommendations for neonates considering only one or two of these variables. For example, Micromedex NeoFax recommends dosing based on a combination of PMA and PNA, pediatric and neonatal Lexicomp recommends two different dosing strategies, one based on a combination of PMA and PNA and the other based on a combination of GA and Scr (Table 1). As a result, the daily doses can differ widely by up to 50% between these studies. Studies have reported that the probability of achieving vancomycin therapeutic targets in neonates with these empiric dosing regimens is only 20%–50% (37).

To date, only two studies with very small sample size have evaluated the optimal vancomycin dosing in VLBW neonates (16, 17). Kato et al. (16) recommended a vancomycin dose of 10 mg/kg every 8 h as the initial dosage regimen for vancomycin in VLBW neonates. This model predicted vancomycin therapeutic target achievement in 86.7% of neonates. However, they developed their model based on data from only 10 VLBW neonates, and the simulations were not divided into categories according to Scr and PMA. The major difference between our findings and the Kato et al. model is that for most groups, except PMA > 29 weeks and low Scr < 0.6 mg/dl, we recommend against three times daily dosing, which would lead to elevated trough levels >20 µg/ml, possibly increasing the risk of nephrotoxicity. Sasano et al. (17) constructed a population pharmacokinetic model for vancomycin dosing based on data from 19 VLBW neonates and infants. They recommended a dose of 5–7.5 mg/kg/doseevery 12 h, especially when Scr > 0.6 mg/dl. The doses recommended by Sasano et al. are lower, compared with our results and those of other references, that is mainly because they evaluated a lower target range of AUC_0−24_ 267–467 µg.h/ml. In our simulations, the selected doses largely depended on the target (AUC_0−24_ range of 400–600 or 400–800 µg.h/ml). Ideally, for each dose, target attainment should be approximately90%, and the probability of achieving toxic concentrations should be <10% (38). However, this would be challenging given the high variability in vancomycin pharmacokinetics in VLBW neonates and the narrow target for vancomycin (AUC_0−24_ 400–600 µg.h/ml). When using the wider target of AUC_0−24_ 400–800 µg.h/ml, dosing regimens could achieve the therapeutic target in 80–90% of patients. The upper cutoff of AUC_0−24_ 800 µg.h/ml was based on a large previous study by Le et al. (9) in a pediatric population.

Notably, the pharmacokinetic/pharmacodynamic (PK/PD) target of vancomycin is mainly based on studies in adults extrapolated to pediatric and neonatal patients. In neonates, clinical studies identifying PK/PD targets and toxicity thresholds are lacking. Nephrotoxicity incidence in neonates is low (0.03%–6%) (39–41). Madigan et al. (41) identified probable vancomycin-related nephrotoxicity in only two out of 57 VLBW neonates. Some studies have attempted to assess the correlation between vancomycin exposure (trough and/or AUC) and nephrotoxicity; however, causal relationships are difficult to identify given the small number of events in these studies. Bhargava et al. (39) found a significant correlation between vancomycin trough concentration and nephrotoxicity. However, only three out of 110 subjects developed nephrotoxicity in this study. On the other hand, Viel-Thériault et al. (42) noted nephrotoxicity in 6% of patients, but the authors could not identify a correlation between vancomycin trough and nephrotoxicity. In a study by Tang et al.(43), increasing AUC_0−24_ was associated with an increase in nephrotoxicity. The authors identified a cutoff of AUC_0−24 _> 485 µg.h/ml as a predictor of nephrotoxicity in neonates. However, only seven out of 182 neonates developed nephrotoxicity in this study. Given the small number of nephrotoxicity events in these studies, it was difficult to draw meaningful conclusions regarding the correlation between vancomycin exposure and nephrotoxicity.

To the best of our knowledge, this is the largest study to evaluate vancomycin pharmacokinetics in neonates with VLBW. However, the limitations of our study include its retrospective nature; all data were collected from one country, and the pharmacokinetic model was built using only 1–2 samples per patient. Therefore, it is important for any center to validate any model to ensure that it fits the patient population before implementation in practice, as our model might not necessarily be generalizable to other populations. The evaluated targets were mainly based on data from adults, not neonates. Our study included only a few patients with Scr > 1.2 mg/dl; hence, our model does not apply to this population. Our study did not evaluate the clinical outcome for the patients. It is important for future studies to assess the correlation between vancomycin concentration and microbiologic and clinical outcome.

In conclusion, our population-specific model-based dosing approach shows promise for improving vancomycin target attainment in neonates with VLBW. Prospective clinical studies are needed to evaluate the benefits of this dosing regimen.

## Data Availability

The raw data supporting the conclusions of this article will be made available by the authors, without undue reservation.

## References

[1] Oza S Lawn JE Hogan DR Mathers C Cousens SN. Neonatal cause-of-death estimates for the early and late neonatal periods for 194 countries: 2000-2013. Bull World Health Organ. (2015) 93(1):19–28. 10.2471/BLT.14.139790 25558104 PMC4271684

[2] Liu L Johnson HL Cousens S Perin J Scott S Lawn JE. Global, regional, and national causes of child mortality: an updated systematic analysis for 2010 with time trends since 2000. Lancet. (2012) 379(9832):2151–61. 10.1016/S0140-6736(12)60560-1 22579125

[3] Hornik CP Fort P Clark RH Watt K Benjamin DK Jr Smith PB. Early and late onset sepsis in very-low-birth-weight infants from a large group of neonatal intensive care units. Early Hum Dev. (2012) 88(Suppl 2):S69–S74. 10.1016/S0378-3782(12)70019-1 22633519 PMC3513766

[4] Sosnin N Curtis N Cranswick N Chiletti R Gwee A. Vancomycin is commonly under-dosed in critically ill children and neonates. Br J Clin Pharmacol. (2019) 85(11):2591–8. 10.1111/bcp.14084 31378957 PMC6848905

[5] Stoll BJ Hansen N Fanaroff AA Wright LL Carlo WA Ehrenkranz RA. Late-onset sepsis in very low birth weight neonates: the experience of the NICHD neonatal research network. Pediatrics. (2002) 110(2 Pt 1):285–91. 10.1542/peds.110.2.285 12165580

[6] Lodise TP Scheetz M Carreno JJ Chambers H Fowler V Holland TL. Associations between vancomycin exposure and acute kidney injury within the recommended area under the curve therapeutic exposure range among patients with methicillin-resistant. Open Forum Infect Dis. (2022) 9(2):ofab651. 10.1093/ofid/ofab651 35079599 PMC8783632

[7] Chavada R Ghosh N Sandaradura I Maley M Van Hal SJ. Establishment of an AUC. Antimicrob Agents Chemother. (2017) 61(5):e02535-16. 10.1128/AAC.02535-16 28242672 PMC5404579

[8] Aljefri DM Avedissian SN Rhodes NJ Postelnick MJ Nguyen K Scheetz MH. Vancomycin area under the curve and acute kidney injury: a meta-analysis. Clin Infect Dis. (2019) 69(11):1881–7. 10.1093/cid/ciz051 30715208 PMC6853683

[9] Le J Ny P Capparelli E Lane J Ngu B Muus R. Pharmacodynamic characteristics of nephrotoxicity associated with vancomycin use in children. J Pediatric Infect Dis Soc. (2015) 4(4):e109–116. 10.1093/jpids/piu110 26582878 PMC4681388

[10] Rybak MJ Le J Lodise TP Levine DP Bradley JS Liu C. Therapeutic monitoring of vancomycin for serious methicillin-resistant Staphylococcus aureus infections: a revised consensus guideline and review by the American society of health-system pharmacists, the infectious diseases society of America, the pediatric infectious diseases society, and the society of infectious diseases pharmacists. Am J Health Syst Pharm. (2020) 77(11):835–64. 10.1093/ajhp/zxaa036 32191793

[11] Marqués-Miñana MR Saadeddin A Peris JE. Population pharmacokinetic analysis of vancomycin in neonates. A new proposal of initial dosage guideline. Br J Clin Pharmacol. (2010) 70(5):713–20. 10.1111/j.1365-2125.2010.03736.x 21039765 PMC2997311

[12] Frymoyer A Hersh AL El-Komy MH Gaskari S Su F Drover DR. Association between vancomycin trough concentration and area under the concentration-time curve in neonates. Antimicrob Agents Chemother. (2014) 58(11):6454–61. 10.1128/AAC.03620-14 25136027 PMC4249374

[13] Abdulla A Edwina AE Flint RB Allegaert K Wildschut ED Koch BC. Model-informed precision dosing of antibiotics in pediatric patients: a narrative review. Front Pediatr. (2021) 9:624639. 10.3389/fped.2021.624639 33708753 PMC7940353

[14] Chow TC Li JY Wong JC Poon FM Lam HS Lam TT. Vancomycin prescribing practices and therapeutic drug monitoring for critically ill neonatal and pediatric patients: a survey of physicians and pharmacists in Hong Kong. Front Pediatr. (2020) 8:538298. 10.3389/fped.2020.538298 33330263 PMC7734090

[15] Li ZL Liu YX Jiao Z Qiu G Huang JQ Xiao YB. Population pharmacokinetics of vancomycin in Chinese ICU neonates: initial dosage recommendations. Front Pharmacol. (2018) 9:603. 10.3389/fphar.2018.00603 29997498 PMC6029141

[16] Kato H Hagihara M Nishiyama N Koizumi Y Mikamo H Matsuura K. Assessment of optimal initial dosing regimen with vancomycin pharmacokinetics model in very low birth weight neonates. J Infect Chemother. (2017) 23(3):154–60. 10.1016/j.jiac.2016.11.009 28017667

[17] Sasano H Aoki K Arakawa R Hanada K. Population pharmacokinetic analysis and dose regimen optimization in Japanese infants with an extremely low birth weight. Antimicrob Agents Chemother. (2021) 65(3):e02523-20. 10.1128/AAC.02523-20 33318009 PMC8092535

[18] Abouelkheir M Almohaizeie A Almutairi A Almuhisen S Alqahtani S Alsultan A. Evaluation of vancomycin individualized model-based dosing approach in neonates. Pediatr Neonatol. (2022). 10.1016/j.pedneo.2022.10.006. [Epub ahead of print] 36581523

[19] Stone SB Benner K Utley A MacLennan P Coghill CH. Achieving vancomycin troughs within goal range in low birth weight neonates. J Pediatr Pharmacol Ther. (2021) 26(1):56–61. 10.5863/1551-6776-26.1.56 33424501 PMC7792141

[20] Vancomycin.. In: Lexicomp Online, Pediatric & Neonatal Lexi-Drugs. Hudson, OH: Lexi-Comp, Inc. (2021). Available from: www.online.lexi.com (cited January 15, 2023).

[21] Vancomycin.. In: IBM Micromedex Neofax. Greenwood Village, CO: IBM Corportation. (2021). Available from: www.micromedexsolutions.com (cited January 15, 2023).

[22] Srivastava T Alon US Althahabi R Garg U. Impact of standardization of creatinine methodology on the assessment of glomerular filtration rate in children. Pediatr Res. (2009) 65(1):113–6. 10.1203/PDR.0b013e318189a6e8 18703997

[23] Anderson BJ Holford NH. Mechanistic basis of using body size and maturation to predict clearance in humans. Drug Metab Pharmacokinet. (2009) 24(1):25–36. 10.2133/dmpk.24.25 19252334

[24] Anderson BJ Allegaert K Holford NH. Population clinical pharmacology of children: modelling covariate effects. Eur J Pediatr. (2006) 165(12):819–29. 10.1007/s00431-006-0189-x 16807729

[25] Anderson BJ Allegaert K Van den Anker JN Cossey V Holford NH. Vancomycin pharmacokinetics in preterm neonates and the prediction of adult clearance. Br J Clin Pharmacol. (2007) 63(1):75–84. 10.1111/j.1365-2125.2006.02725.x 16869817 PMC2000709

[26] Holford N. Dosing in children. Clin Pharmacol Ther. (2010) 87(3):367–70. 10.1038/clpt.2009.262 20090674

[27] Brendel K Comets E Laffont C Mentré F. Evaluation of different tests based on observations for external model evaluation of population analyses. J Pharmacokinet Pharmacodyn. (2010) 37(1):49–65. 10.1007/s10928-009-9143-7 20033477 PMC2874574

[28] Le J Bradley JS Murray W Romanowski GL Tran TT Nguyen N. Improved vancomycin dosing in children using area under the curve exposure. Pediatr Infect Dis J. (2013) 32(4):e155–63. 10.1097/INF.0b013e318286378e 23340565 PMC3632448

[29] Lodise TP Patel N Lomaestro BM Rodvold KA Drusano GL. Relationship between initial vancomycin concentration-time profile and nephrotoxicity among hospitalized patients. Clin Infect Dis. (2009) 49(4):507–14. 10.1086/600884 19586413

[30] van Hal SJ Paterson DL Lodise TP. Systematic review and meta-analysis of vancomycin-induced nephrotoxicity associated with dosing schedules that maintain troughs between 15 and 20 milligrams per liter. Antimicrob Agents Chemother. (2013) 57(2):734–44. 10.1128/AAC.01568-12 23165462 PMC3553731

[31] Neely MN Youn G Jones B Jelliffe RW Drusano GL Rodvold KA. Are vancomycin troughs adequate for optimal dosing? Antimicrob Agents Chemother. (2013) 58:309–16. doi: 10.1128/AAC.01653-13 10.1128/AAC.01653-13 PMC3910745 24165176

[32] Alsultan A Abouelkheir M Albassam A Alharbi E Assiri A Alqahtani S. AUC- vs. trough-guided monitoring of vancomycin in infants. Indian J Pediatr. (2020) 87(5):359–64. 10.1007/s12098-019-03162-5 31984471

[33] Moise-Broder PA Forrest A Birmingham MC Schentag JJ. Pharmacodynamics of vancomycin and other antimicrobials in patients with Staphylococcus aureus lower respiratory tract infections. Clin Pharmacokinet. (2004) 43(13):925–42. 10.2165/00003088-200443130-00005 15509186

[34] Rybak MJ Le J Lodise TP Levine DP Bradley JS Liu C. Therapeutic monitoring of vancomycin for serious methicillin-resistant Staphylococcus aureus infections: a revised consensus guideline and review by the American society of health-system pharmacists, the infectious diseases society of America, the pediatric infectious diseases society, and the society of infectious diseases pharmacists. Clin Infect Dis. (2020) 71(6):1361–4. 10.1093/cid/ciaa303 32658968

[35] Men P Li HB Zhai SD Zhao RS. Association between the AUC0-24/MIC ratio of vancomycin and its clinical effectiveness: a systematic review and meta-analysis. PLoS One. (2016) 11(1):e0146224. 10.1371/journal.pone.0146224 26731739 PMC4701440

[36] Aljutayli A El-Haffaf I Marsot A Nekka F. An update on population pharmacokinetic analyses of vancomycin, part II: in pediatric patients. Clin Pharmacokinet. (2022) 61(1):47–70. 10.1007/s40262-021-01050-w 34671937

[37] Frymoyer A Stockmann C Hersh AL Goswami S Keizer RJ. Individualized empiric vancomycin dosing in neonates using a model-based approach. J Pediatric Infect Dis Soc. (2019) 8(2):97–104. 10.1093/jpids/pix109 29294072

[38] Turnidge J Paterson DL. Setting and revising antibacterial susceptibility breakpoints. Clin Microbiol Rev. (2007) 20(3):391–408. table of contents. 10.1128/CMR.00047-06 17630331 PMC1932754

[39] Bhargava V Malloy M Fonseca R. The association between vancomycin trough concentrations and acute kidney injury in the neonatal intensive care unit. BMC Pediatr. (2017) 17(1):50. 10.1186/s12887-017-0777-0 28187757 PMC5303210

[40] Alrahahleh D Xu S Luig M Kim HY Alffenaar JW. Dosing of vancomycin and target attainment in neonates: a systematic review. Int J Antimicrob Agents. (2022) 59(2):106515. 10.1016/j.ijantimicag.2021.106515 35031450

[41] Madigan T Teng CB Koshaish J Johnson KR Graner KK Banerjee R. Optimization of vancomycin dosing in very low-birth-weight preterm neonates. Am J Perinatol. (2015) 32(1):83–6. 10.1055/s-0034-1376183 24839147 PMC4418186

[42] Viel-Thériault I Martin B Thompson-Desormeaux F Blackburn J Moussa A Autmizguine J. Vancomycin drug monitoring in infants with CoNS sepsis-target attainment, microbiological response and nephrotoxicity. J Perinatol. (2020) 40(1):97–104. 10.1038/s41372-019-0519-2 31576000

[43] Tang Z Guan J Li J Yu Y Qian M Cao J. Determination of vancomycin exposure target and individualised dosing recommendations for neonates: model-informed precision dosing. Int J Antimicrob Agents. (2021) 57(3):106300. 10.1016/j.ijantimicag.2021.106300 33567334

